# Ulysses - an application for the projection of molecular interactions across species

**DOI:** 10.1186/gb-2005-6-12-r106

**Published:** 2005-12-02

**Authors:** Danielle Kemmer, Yong Huang, Sohrab P Shah, Jonathan Lim, Jochen Brumm, Macaire MS Yuen, John Ling, Tao Xu, Wyeth W Wasserman, BF Francis Ouellette

**Affiliations:** 1 Center for Genomics and Bioinformatics, Karolinska Institutet, 171 77 Stockholm, Sweden; 2 Centre for Molecular Medicine and Therapeutics, University of British Columbia, Vancouver V5Z 4H4, BC, Canada; 3 UBC Bioinformatics Centre, University of British Columbia, Vancouver V6T 1Z4, BC, Canada; 4 Department of Medical Genetics, University of British Columbia, Vancouver, BC, Canada; 5 Michael Smith Laboratories, University of British Columbia, Vancouver V6T 1Z4, BC, Canada; 6 Department of Computer Science, University of British Columbia, Vancouver V6T 1Z4, BC, Canada

## Abstract

We developed Ulysses as a user-oriented system that uses a process called Interolog Analysis for the parallel analysis and display of protein interactions detected in various species. Ulysses was designed to perform such Interolog Analysis by the projection of model organism interaction data onto homologous human proteins, and thus serves as an accelerator for the analysis of uncharacterized human proteins. The relevance of projections was assessed and validated against published reference collections. All source code is freely available, and the Ulysses system can be accessed via a web interface .

## Abstract 2

Ulysses, a new software for the parallel analysis and display of protein interactions detected in various species, is described.

## Rationale

The catalogue of human protein-encoding genes is largely enumerated [1], but the task of discerning the functions of these genes remain a formidable challenge. A significant fraction of protein-encoding genes are entirely novel; the cellular roles of the proteins remain a mystery. As model organism genome sequences have been available for several years, a modest compendium of functional genomics data has emerged for these organisms. To capitalize on these data for the functional annotation of human genes, one can project model organism gene properties onto homologous human genes [2]. Although the properties of homologous genes are often predicted based on recorded annotations of genes with similar sequences, such mappings only begin to capitalize on available data.

The increasing body of genomics data allows functions to be predicted using 'Guilt by Association' (GBA) methods. In GBA, the function of a gene is inferred from the functions of genes with which it interacts (for example, protein contact) or parallels (for example, co-expression). Observation of mutually consistent interactions in multiple species improves the predictive performance of GBA methods, a process named Interolog Analysis [2,3]. Early demonstrations of the utility of Interolog Analysis, although limited to the analysis of model organism data, offer promise for the accelerated annotation of human genes.

Prediction of human gene function based on Interolog Analysis requires an underlying set of bioinformatics resources and algorithms to make unified data accessible to the community. First, functional genomics data must be accessible through reference databases. Second, the relationships between homologous genes must be mapped by a suitable comparison procedure. Finally, the relationships must be rendered accessible to the broad community through an intuitive interface. A system incorporating these three components would be a powerful tool for laboratory investigators seeking to capitalize on existing genomics data.

Despite substantial success in sequencing genomes, large-scale functional studies have been reported for only a few common model organisms. Key reports have addressed protein-protein interactions in *Saccharomyces cerevisiae *[4-6], *Drosophila melanogaster *[7-9], and *Caenorhabditis elegans *[10]. In addition to these screens, functional studies have linked genes by tackling such topics as: patterns of co-expression [11], genetic interactions [12], and sub-cellular co-localization [13]. The diverse data from the functional studies have been rendered publicly accessible in species-specific repositories [14-16]. Large databases that have emerged to consolidate the diverse functional genomics data include leading examples like the Biomolecular Interaction Network Database (BIND) [17], DIP [18], and MINT [19].

To manage the combination of interaction data and genome annotation, data warehouses have emerged such as EnsMart [20], SeqHound [21], and Atlas [22]. All three examples store heterogeneous biological data in a relational schema, allowing for rapid retrieval using Structured Query Language (SQL) via an integrated application programming interface (API), or via a web graphical user interface.

In order to draw conclusions about human genes from model organism data, it is essential to possess a map enumerating gene homology relationships among species. The fundamental assumption is that direct gene orthologs (genes separated only by speciation) typically occupy the same functional niche [23]. Leading systems such as COGs [24,25] and Inparanoid [26] continue to unravel the complex evolutionary relationships between genes. As shown by these efforts, the stringent demands for orthology mapping are challenging, so it is often more feasible to group homologs. The National Center for Biotechnology Information's (NCBI) HomoloGene [27] provides such a high-throughput map suitable for incorporation into larger analyses that address many organisms. The establishment of evolutionary relationships between genes remains a topic of active investigation.

Biological interpretation of integrated data is greatly aided by tools for visualization of properties. Multiple platforms for the visualization and manipulation of protein interaction networks [28-32] provide users with interfaces to complex interaction data. Interolog Analysis has emerged as a powerful means to predict the function of genes [2,33-36]. Existing Interolog Analysis tools, like the Interolog database [3] and STRING [37], convey information about protein associations across species using databases, homology maps, and simple visualization methods. These visualization tools, however, are restricted to single views that fail to convey the evidence from each species.

**Figure 1:**
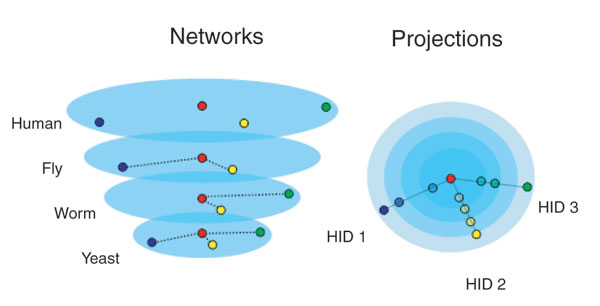
Interologs mapping of conserved protein networks across multiple species (each plane corresponds to a species). Orthologous proteins are defined and protein interactions identified in each model organism. Virtual human protein networks are generated by projecting the observed interactions across all planes onto homologous human genes. HID, HomoloGene identifier.

We report the construction and assessment of a novel Interolog system for the exploration of human genes based on gene-gene interactions in yeast, fly, and worm (Figure 1). The system displays composite interaction networks composed of protein associations detected in the model organisms. The system unites the Atlas database, HomoloGene mappings, and a new Interolog visualization tool, all accessed via a user-friendly web interface entitled Ulysses [38]. We assessed the performance of the underlying Interolog algorithm against published reference collections of protein interactions, revealing a statistically significant ability to link genes to the correct networks. Redundantly observed gene-gene associations across datasets or species are demonstrated to be remarkably specific. We applied the most accurate parameters to predict human protein interactions and new candidate members for inclusion in known pathways and complexes.

## Model organism data to predict human protein interactions

The available pool of curated annotations of protein-protein interactions in reference databases is sparse, only a small subset of the interactome (the complete collection of all functionally relevant protein-protein interactions) is present. The Human Protein Reference Database (HPRD) [39] is the largest curated collection of documented human protein interactions. To assess the relevance of observed interactions between model organism proteins for the prediction of human interactions, we determined the overlap between protein interactions in the HPRD reference dataset and homologous interactions from model organisms represented in BIND [17]. Reflecting the sparse coverage of the interactome, only 80 such interactions were found. The sparse coverage of *bona fide *protein-protein interactions is problematic to evaluating the performance of predictive methods. Previous studies have assessed the quality of interaction data on the basis of protein interactor pairs sharing the same annotated GO-terms [33,35,40,41] or pathway assignments [42]. While such measures are often supportive of the predictive performance of methods, we believe such criteria suffer from a focus on the strongest and most easily observed interactions.

To gain a broader assessment of the relevance of mapping interactions from model organism proteins onto corresponding homologous human proteins, we elected to apply a compartment-based assessment of the Interolog Analysis. As protein interactions preferentially occur between proteins residing in the same sub-cellular compartment [13,43], interactions between two proteins were considered to be true if both interactors co-localized to the same sub-cellular location. To validate this approach, we analyzed yeast interactions reported in BIND that have an annotated Gene Ontology (GO) [44] localization label. We distinguished between low-throughput (LTP: less than 40 interaction records in the same publication, using the same experimental method) and high-throughput (HTP) data and counted interactions supported by at least two independent reports (Table 1). For LTP and HTP experiments, respectively, 79% and 77% of the interactors from the redundantly observed interactions matched major sub-cellular compartments (nucleus, cytoplasm, extra-cellular space), both statistically significant in comparison to background levels. Exact matches to highly specific GO compartments were 59% for LTP and 24% for HTP data. This difference at the specific compartment level reflects the tendency for well-studied genes (those that have been the focus of LTP studies) to be deeply annotated. Given the correlation between interaction and general sub-cellular localization of yeast proteins, we adopted the criterion of co-localization to assess the predictive value of Interolog Analysis for the study of human protein interactions.

**Figure 2:**
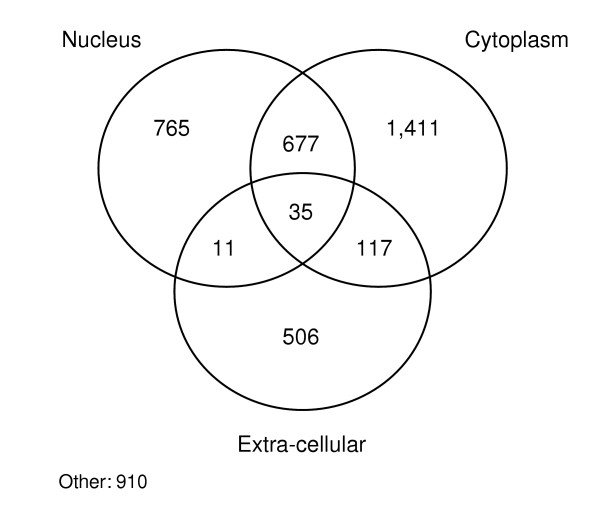
Distribution of RefSeq/HomoloGene proteins across HPRD cellular localization bins. Protein interactors from BIND were mapped to HomoloGene to delineate homologs across the four organisms, and to associate each protein to a sub-cellular compartment.

We mapped all human RefSeq identifiers for proteins in the HPRD database (6,141 proteins) to HomoloGene identifiers (5,308 HomoloGene groups). Each HomoloGene interactor was assigned to one or more cell compartment(s) based on the curated HPRD annotations (Table 2). As a control data set for the rate of co-localization for arbitrary pairs of interactors, we randomly created 60,000 pairings of the HomoloGene groups represented in the HPRD data. HomoloGene identifiers were retrieved for *S. cerevisiae*, *D. melanogaster*, and *C. elegans *proteins reported as interactors in the BIND database. For each model organism interactor mapping to the same HomoloGene as an HPRD human protein, the sub-cellular compartment (as defined by HPRD) was noted (Figure 2). For 28,254 interactions, both interactors were annotated as localizing within at least one cellular compartment (Table 3). In a second step, for each of these pairs, we determined if both protein interactors co-localized to the same cellular location, that is, if they shared at least one cellular compartment. For BIND-reported interacting pairs, co-localization was true for between 75% and 97%, depending on the species and method (Table 4). Compared to the background rate of 66% for the randomly generated pairs of interactors (which reflects the fact that many proteins are annotated with multiple localizations), every category was significantly biased towards co-localization. The success rates for yeast two-hybrid (Y2H) data reached 87% in worm, but only 75% in fly. This observation agrees with a recent study [33], where the authors attributed greater confidence to protein interactions originating from the published HTP experiments for *S. cerevisiae *and *C. elegans *compared to the published results for *D. melanogaster*.

To identify predictions of greater specificity, we determined the co-localization rates for proteins for which 'double linkage' interactions were observed, where 'double linkage' refers to interactions supported either by two different experimental methods for a single organism or in data from two different species (Table 5). As for single linkage interactions, the background co-localization rate for randomly selected pairs of interactors was 66%. For those interacting pairs with double linkage in BIND, 100% co-localization was observed. Even though our results were concordant with earlier reports [3,33,43], the number of 'double linkage' interactions (n = 4 to 28) was too sparse to achieve statistical significance, but the perfect predictive specificity is qualitatively noteworthy.

## Negative control data

Because a curated reference collection of non-interacting human proteins is lacking and because pairs of proteins residing in different sub-cellular compartments are less likely to interact [45], we assessed the noise in the interaction data by the frequency with which HomoloGene interactors were annotated with incompatible localizations. We evaluated proteins localizing to the nucleus, the cytoplasm, and the extra-cellular space. We considered all model organism protein interactions for which both interactors mapped to a HomoloGene containing a human protein with annotated localization in the HPRD database. We found that 'true' interactions, that is, interactions between two model organism proteins annotated with the same compartment, accounted for 91% and inconsistencies were observed in 9% of the cases. As proteins can exist in different compartments at different times, and the curated HPRD annotations are restricted to the available literature, the inconsistencies should be viewed as an upper-bound of the false classification rate. It is noteworthy that there were no inconsistencies for the double linkage interactions.

## Network expansion and detection (multi-protein interactions)

KEGG [46] and PINdb [47] are curated annotation databases describing biological pathways and complexes. To demonstrate the capacity of Ulysses to detect new components of these known pathways and complexes, we identified candidates based on the following double linkage criteria: the candidate interacted with two or more pathway members in one organism; or the candidate interacted with homologous proteins of pathway members in two or more species. Based on these criteria and after mapping all pathway and complex components to HomoloGene, 14 HomoloGenes were newly associated with 11 pathways and complexes previously described in KEGG and PINdb (Additional data file 1). Several of these candidates have been previously linked to the pathways or processes in the scientific literature, but have not yet been annotated as such in the reference databases.

Based on the ability of the Ulysses system to identify candidates for inclusion in known networks, we sought to uncover interconnected networks within which each member is connected to at least two other members. Extracting all pairs of HomoloGene proteins supported by two or more datasets, for which there was at least one human homolog for each interactor, we were able to identify 127 distinct HomoloGenes involved in 82 interactions. Amongst these observed high confidence pairwise interactions (Table 6 and Additional data file 2) were two apparently novel interactions involving disease-linked genes. The *YEATS4 *gene, a poorly characterized gene known as glioma-amplified sequence 41, was linked to DMAP1, a DNA methyltransferase-associated protein. The *DGCR14 *gene from the DiGeorge Syndrome critical region was found to interact with VDP, a vesicle docking protein linked to the golgi. Table 6 specifies candidate interactions for which we could not identify existing support, while Additional data file 2 lists those interactions that appear consistent with established literature.

Grouping of overlaps in these high confidence interactions revealed previously characterized networks, including highly conserved pathways and complexes.

We recovered elements of the spliceosome, including seven core small nuclear ribonucleoprotein particle (snRNP) components (LSM1, 2, 4, 5, 7, 8, SNRPD2), four U2 and U3 snRNP-specific proteins (SF3A3, IMP3, IMP4, MPHOSPH10), a splicing factor (PRPF19), as well as a protein usually associated with the PRPF19 complex (CRNKL1) known to interact with the spliceosome [48].

Two clusters were observed composed of proteins required for DNA replication and repair, as well as replication-dependent structural proteins. One cluster contained all five subunits (RFC1, 2, 3, 4, 5) of an accessory factor for DNA replication, replication factor C (RF-C). The other cluster contained four nucleosomal proteins, three members of the H2A histone family (H2AFE, H2AFJ, H2AFN), which were all connected to the nucleosome assembly protein 1-like 1 (NAP1L1).

We also identified a network of 19 interconnected proteasome subunits. We found five core alpha (PSMA1, 2, 3, 5, 7) and four core beta subunits (PSMB3, 4, 5, 7) from the 20S proteasome, as well as nine subunits from the 19S regulatory complex. We located the proteasome regulatory particle subunit PSMD6 interacting with PSMD3, a non-ATPase subunit of the 19S regulatory complex.

These examples of functional networks among protein members of well conserved cellular complexes and pathways validate our approach to detect biologically meaningful protein interactions in human by overlaying and projecting interaction data originating from diverse model organisms.

To date, the limiting factor for network discovery is the sparse protein interaction data. As more association data are generated for the core model organisms, the Ulysses Interolog analysis system will facilitate greater inference of network members.

## Ulysses web interface for analysis and visualization of networks

**Figure 3:**
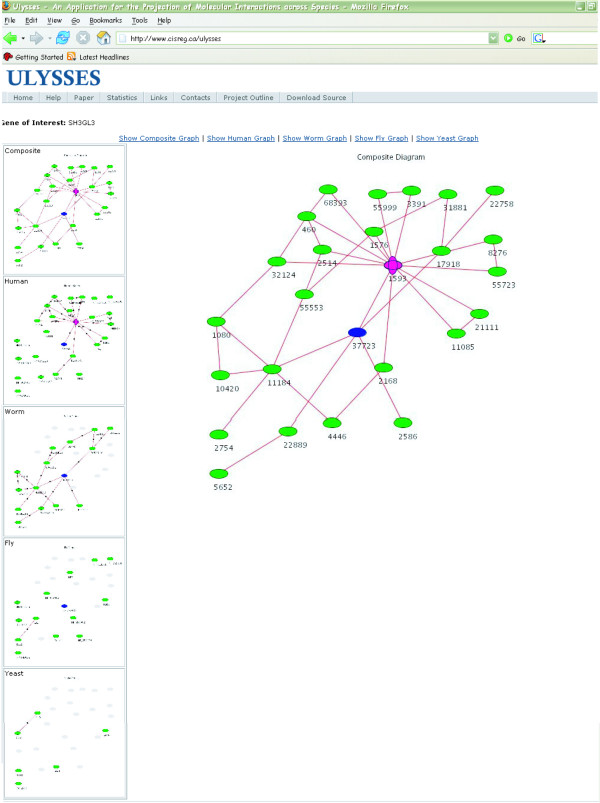
Screenshot of the Ulysses interface. The user-specified protein is shown in blue, and interacting proteins are displayed in green. Proteins with greater than three interactions (the 'starburst' threshold) are marked with a magenta-colored cross. The colors and 'starburst' threshold are user-adjustable parameters. Species-specific interactions are displayed in the panel of windows on the left. In this figure, the central graph displays a composite image identifying each node with its HomoloGene identifier. By selecting a species window, the species-specific interactions will be displayed along with the identity of the individual protein interactors.

To bring the power of multi-organism network analysis to laboratory researchers, a web-based interface to the Ulysses system was implemented [38] (Figure 3). A user enters the database with a gene of interest by submitting either the gene name or symbol, an accession ID, or even by pasting the protein sequence of the corresponding gene product. The system calls to the Atlas database and returns all interactions reported in the BIND database for homologous proteins in the model organisms, as well as the secondary interactions to the direct partners of the reference gene. These primary and secondary interactors are plotted and displayed in a series of network windows for each species. The option to individually display species-specific protein networks allows the user to trace back the origin of the projected data; the user can assess projections based on the source of evidence. The user can further choose to display a composite image overlaying interaction data for homologous genes in all organisms, or limit the view to an individual species. The original protein of interest and its homologs are clearly labeled across all organisms. In each display mode, 'starburst' proteins, defined as proteins involved in excess of a user-defined number of interactions, are color-coded and easily identified (such 'starbursts' may represent genes prone to false interactions in HTP studies). These 'starbursts' can be displayed in either a compacted fashion or expanded. Individual protein interactions are linked to publications citing the corresponding association. The database also links each gene in organism-specific networks to gene information in external resources such as GeneLynx [49], SGD [16], WormBase [15], and FlyBase [14].

## Utility and comparison to other systems

Here we described an exploratory Interolog Analysis framework for the inference of protein function. We demonstrate, by overlaying protein interaction data sets, dramatic improvements in the specificity of projected 'dual-linkage' interactions compared to those based on a single study. Through a novel interface, we provide a means for the broad community of researchers to use Interolog Analysis for the directed study of specific pathways or processes.

Ulysses represents a significant advance in the graphical display of protein interaction data for comparative genomics. Visualization tools for the study of protein and genetic networks have been available for many years, including Cytoscape [32], Osprey [31], and ProViz [28]. These useful tools have enabled researchers to display networks for a single species or data set. Each of these tools requires submission of a pre-computed table of results, whereas Ulysses both performs the data analysis and renders a visual display. To our knowledge, only two software tools provide interfaces for comparative analysis of protein interactions (Interolog Analysis). POINT [36] displays pairwise network diagrams; however, positions of homologous proteins are not preserved between panes, making visual interpretation exceedingly difficult. The mature STRING system [37] features an excellent underlying data collection. The STRING visual interface for comparative analysis, however, is restricted to a composite plot - there is no parallel display for individual species. Although the underlying data in STRING is robust, only the most advanced users of the system can extract the information provided intuitively in the Ulysses interface. Thus Ulysses is unique in its capacity for parallel display of interaction data from multiple species for comparative analysis and biological interpretation.

A limiting factor for inference of new protein clusters and extension of known clusters is the sparse existing coverage of interactions in genomics data. Even though proteome-scale analyses have been conducted for several organisms [4,7,10], the lack of overlapping interactions limits the impact of the analysis of interactions shared by homologs. In this study, we found that interactions observed in multiple studies (for homologous proteins) are highly reliable (Table 5). As more extensively overlapping interaction data sets emerge, Interolog Analysis will allow for expanded functional annotation of human genes. Individual uncharacterized genes will be linked to known cellular pathways and complexes, and we anticipate the discovery of new functional units. To this end, we strongly encourage protein interaction screens of additional organisms and deeper coverage of the primary model organisms, as the depth of data is critical to increasing the utility of Interolog Analysis.

The homology mapping obtained from HomoloGene was convenient for the Ulysses system. Because homology mapping across organisms remains an issue of debate, however, future releases of Ulysses will offer an option to choose between different resources, possibly including well established systems [24,26,27].

Even though the small size of the present body of functional genomics data does not allow for extended *de novo *discovery of cellular networks, detection of known complexes and pathways demonstrate Ulysses' capacity to successfully identify biological networks. Ulysses is available without restriction as an internet-based resource or as downloadable code for developers [38]. The novel interface partitions data into discrete planes, offering an intuitive means of performing Interolog Analysis.

## Materials and methods

### Database implementation

All data were stored within the Atlas database system [22,50]. The Atlas data warehouse provides a framework for integrating data from diverse systems within a unified environment. All data sets were imported from indicated databases using the SQL interface or Java API. All software and scripts used to extract data from the Atlas system are available by request.

### Interaction data

Protein interaction data were obtained from BIND [51] (freeze August 2004). Direct protein-protein interactions from yeast two-hybrid experiments and indirect associations from protein complex purification experiments were extracted. Table 7 reports the number of unique interactions and interactors (proteins) acquired for each method and model organism. For the online system, protein interaction data from BIND are updated automatically. At the time of publication, the interaction data underlying the Ulysses system were updated as of October 2005.

### Homology mapping

#### HomoloGene

HomoloGene [52] is an NCBI resource providing computationally identified homologs to human protein reference sequences derived from the RefSeq collection [53]. We used data from HomoloGene freeze July 2004, which included 26,797 HomoloGene groups and 108,734 unique genes. The HomoloGene dataset was seeded by a non-redundant human RefSeq protein sequence collection and compared using protein-protein BLAST [54] to RefSeq protein sequences from model organisms. After mapping the protein sequences back to their respective genomes, both distance (Ka/Ks ratios [55]) and synteny were assessed to identify false pairings.

#### Ortholog mapping for model organisms

For proteins from each of the three included model organisms (worm, fly, and yeast), unique GenBank protein geninfo (gi) numbers were extracted from BIND. These identifiers were mapped to corresponding identifiers in the RefSeq collection and the RefSeq IDs were used to select homology sets in HomoloGene. For BIND sequences without a mapping to a RefSeq sequence, BLAST analysis was performed against a database of all RefSeq sequences represented in the HomoloGene system. Parameters were set to an e-value cutoff of 10^-20^, and sequences were only included in the set if the matching portion included the entirety (100%) of the query sequence. At the time of publication, homology mappings through HomoloGene were updated as of September 2005.

### Reference data sets and evaluation criteria

The HPRD is a collection of hand-curated reports on human proteins extracted from the scientific literature [39]. The HPRD collection (HPRD freeze July 2004: 13,469 proteins, 26,893 protein interactions) was uploaded into the Atlas database, and protein identifiers were mapped to corresponding HomoloGene and RefSeq identifiers. The HPRD annotations include reported sub-cellular locations for each protein.

### Statistical evaluation

#### Interaction data set from model organisms

A total of 32,930 binary and protein complex interactions were obtained from BIND for which both interactors had been successfully mapped to HomoloGene homology groups. These interactions constitute the observed data and were assessed relative to the HPRD reference set.

#### Sampling from HPRD

We generated 60,000 random pairings of all interactors (proteins) present in HPRD bearing a localization label. After eliminating redundancy, we used this set to determine the sub-cellular co-localization. Statistical significance was evaluated using the Fisher exact test.

### Visualization and web interface

The Ulysses visualization system dynamically generates images for display in a web browser. The visualization problem was divided into two tasks: graph network layout and image rendering. The open source JUNG (Java Universal Network/Graph) Framework [56] was used for modeling the network structure, based on interaction data extracted from the Atlas database via the Atlas API. Image rendering and web page generation were performed by a Java framework composed of the following components: JavaServer Pages (JSPs), standard Java libraries included with J2SE 1.5.0 [57], and the Java Advanced Imaging (JAI) libraries [58]. JSPs were used to unite the various components. The visualization application is deployed using the Tomcat web application server [59]. The network layout is defined using all reported HomoloGene sets in all organisms, and the species-specific images are constructed by limiting the display to proteins participating in interactions within the species. This process allows for the positions of homologous genes to be maintained across species.

## Additional data files

The following additional data are available with the online version of this paper. Additional data file 1 is a table showing new HomoloGene associations with known pathways and complexes described in KEGG and PINdb. Additional data file 2 lists the human protein interaction predictions supported by redundant observations for homologous proteins in model organisms.

## Supplementary Material

Additional data file 1 Species include *S. cerevisiae *(Sc), *C. elegans *(Ce), and *D. melanogaster *(Dm). These candidate connections included genes linked to core biological functions. New candidate members were identified for protein degradation processes, including ubiquitin-dependent protein catabolism and protein degradation via the proteasome. Additionally, candidates were linked by interactions to the ribosome, maintenance, and nuclear export. New candidate components were linked to the highly conserved RNA polymerase complex. Finally, novel associations were predicted with membrane and vesicle-based targeting proteins. These examples illustrate the capacity of the Ulysses system to reveal starting points for the study of new complexes or to orient the exploration of new candidate members of known functional units Click here for file

Additional data file 2 Double linkage criteria (see Table 5) revealed high confidence protein associations. Interacting partners 1 and 2 are listed with their human gene symbols and HomoloGene groups Click here for file

## Figures and Tables

**Table 1:** Yeast protein interactions reported in BIND confirmed by co-localization

	Total	Independently confirmed interactions	Bin match	Exact match
Low-throughput	1,753	565	448 (79%)	335 (59%)
High-throughput	54,439	4,485	3,464 (77%)	1,096 (24%)

Data were from BIND freeze 20 April 2005. Bin matches refer to protein interactors localizing to the same major cellular compartments (nucleus, cytoplasm, extra-cellular space). Exact matches refer to specific sub-cellular locations captured by GO annotations.

**Table 2:** Composition of localization bins

Cytoplasm (C)	Nucleus (N)	Extra-cellular (E)	Other
Cytoplasm	Nucleus	Extra-cellular	Plasma membrane
Mitochondria	Nucleolus		Membrane
Endoplasmic reticulum			Centrosome
Golgi apparatus			Other
Lysosome			
Endosome			
Sarcoplasmic reticulum			
Peroxisome			
Ribosome			

Detailed localization labels from HPRD were used to assign each sub-cellular compartment to at least one of the three major cellular localizations. Localization labels that could not be classified were excluded from the analysis (other).

**Table 3:** Data resources for performance evaluation

Source	Interactions
Randomly generated human (HPRD)	59,981*
Model organisms (BIND)	32,930
Total pairs	92,911
Pairs mapped to cellular compartments	28,254

*Redundancy was eliminated from the initial 60,000 random human interactions.

**Table 4:** Cross-classification of interaction and localization - single projections

	Yeast two-hybrid						Complex purification	
Interactions	Yeast		Worm		Fly		Yeast	
	
	Random	BIND	Random	BIND	Random	BIND	Random	BIND
	
No co-localization	9,493	3	9,475	21	9,454	42	9,314	182
Co-localization	18,656	102	18,614	144	18,628	130	17,411	1,347
Total	28,149	105	28,089	165	28,082	172	26,725	1,529
Success rate	66.28	97.14	66.27	87.27	66.33	75.58	65.15	88.10
p-value		4.3e-09		8.67e-08		0.0111		2e-16

Interactions from model organisms reported in BIND for which both interactors could be mapped to human homologs (HPRD) were evaluated for co-localization. Random interactions generated for HPRD interactors are shown as control datasets.

**Table 5:** Cross-classification of interaction and localization - double projections

	Yeast two-hybrid						Yeast two-hybrid/complex purification	
Interactions	Yeast/worm		Yeast/fly		Fly/worm		Yeast	
	
	Random	BIND	Random	BIND	Random	BIND	Random	BIND

No co-localization	9,472	0	9,451	0	9,433	0	9,311	0
Co-localization	18,520	8	18,532	6	18,488	4	17,337	28
Total	27,992	8	27,983	6	27,921	4	26,648	28
Success rate	66.16	100	66.23	100	66.22	100	65.06	100

Double linkages from model organisms for which each interaction was either reported in at least two different species or datasets were evaluated for co-localization. Random interactions between HPRD interactors are displayed for control.

**Table 6:** Human protein interaction predictions supported by redundant observations for homologous proteins in model organisms

HomoloGene ID 1	Gene symbol 1	HomoloGene ID 2	Gene symbol 2
5257	*XAB1*	7006	*ATPBD1B*
6136	*NACA*	932	*LOC391040*
6127	*NMD3*	3139	*COX5A*
5998	*NOL10*	5682	*AATF*
5601	*PSF1*	5759	*SLD5*
5754	*MCEMP1*	5436	*TRAPPC2*
5368	*C20orf14*	5574	*UBE2I*
12733	*MGC4093*	8440	*EPPB9*
1220	*ACTG1*	4643	*LOC401076*
3531	*WDR39*	6115	*CGI-128*
5257	*XAB1*	6487	*ATPBD1C*
5715	*BCCIP*	755	*RPL23*
682	*IMPDH2*	1080	*EIF2B1*
5356	*PP*	1080	*EIF2B1*
20319	*ARL1*	3444	*ATP6V0D1*
1776	*MAGOH*	3744	*RBM8A*
5699	*SKP1A*	4485	*NEDD8*
10422	*DMAP1*	4760	*YEATS4*
2900	*ZNF259*	6872	*RBX1*
10363	*KCTD5*	9180	*GORASP2*
2754	*VDP*	11184	*DGCR14*

Double linkage criteria (see Table 5) revealed high confidence protein associations. Interacting partners 1 and 2 are listed with their human gene symbols and HomoloGene groups. Previously known interactions are reported in Additional data file 2.

**Table 7:** Model organism protein interaction datasets

	Yeast		Fly		Worm	
Source	Interactions	Interactors	Interactions	Interactors	Interactions	Interactors

BIND - Y2H	6,799	3,837	18,899	6,785	5,100	2,907
HomoloGene	2,110	1,562	4,448	2,614	1,639	1,170
BIND - complex	56,109	2,356	8	7	-	-
HomoloGene	24,733	1,530	-	1	-	-

HomoloGene interactions indicate the number of BIND (freeze 4 August 2004) interactions for which both interactors could be mapped to human genes by HomoloGene.
