# Supplementary material for: Ulysses - an application for the projection of molecular interactions across species
Source: Genome Biol. 2005 Dec 2;6(12):R106. doi: 10.1186/gb-2005-6-12-r106 (PMC1414088; doi:10.1186/gb-2005-6-12-r106)
Supplement: Additional data file 1 — Species include S. cerevisiae (Sc), C. elegans (Ce), and D. melanogaster (Dm). These candidate connections included genes linked to core biological functions. New candidate members were identified for protein degradation processes, including ubiquitin-dependent protein catabolism and protein degradation via the proteasome. Additionally, candidates were linked by interactions to the ribosome, maintenance, and nuclear export. New candidate components were linked to the highly conserved RNA polymerase complex. Finally, novel associations were predicted with membrane and vesicle-based targeting proteins. These examples illustrate the capacity of the Ulysses system to reveal starting points for the study of new complexes or to orient the exploration of new candidate members of known functional units [file gb-2005-6-12-r106-S1.doc]

**Supplemental Table 1**. New HomoloGene associations with known pathways and complexes described in KEGG and PINdb. Species include *S. cerevisiae* (Sc), *C. elegans* (Ce), and *D. melanogaster* (Dm). These candidate connections included genes linked to core biological functions. New candidate members were identified for protein degradation processes, including ubiquitin-dependent protein catabolism and protein degradation via the proteasome. Additionally, candidates were identified for interaction with the ribosome, either as structural components or required for assembly, maintenance, and nuclear export. New candidate components were linked to the highly conserved RNA polymerase complex. Finally, novel associations were predicted to membrane and vesicle-based protein targeting. These examples illustrate the capacity of the Ulysses system to reveal starting points and to direct the exploration of additional biological associations with known functional units.

| **Pathway/Complex** | **HomoloGene** | **RefSeq** | **Gene name** | **Species** |
| --- | --- | --- | --- | --- |
| PFD-like complex | 19105 | NP_012623 | GRR1 | Sc |
|  | 4877 | NP_014700 | SGT1 | Sc |
|  |  | NP_505751 | 5L253 | Ce |
|  | 4485 | NP_010423 | RUB1 | Sc |
|  |  | NP_492717 | ned-8 | Ce |
| Oxidative phosphorylation^1^ | 6127 | NP_012040 | NMD3 | Sc |
|  |  | NP_477332 | Nmd3 | Dm |
| Ubiquitin-mediated proteolysis | 2900 | NP_011727 | ZPR1 | Sc |
|  |  | NP_572532 | Zpr1 | Dm |
| Alzheimer’s disease | 2951 | NP_015391 | UBA3 | Sc |
|  |  | NP_610913 | CG13343 | Dm |
| Jak-STAT signaling pathway | 3457 | NP_014403 | VPS27 | Sc |
|  |  | NP_525099 | Hrs | Dm |
| Cholera infection | 20319 | NP_009723 | ARL1 | Sc |
|  |  | NP_524098 | Arf72A | Dm |
| Ribosome | 5715 | NP_010648 | BCP1 | Sc |
|  |  | NP_650317 | CG9286 | Dm |
|  | 6517 | NP_013444 | RPP0 | Sc |
|  |  | NP_524211 | RpLP0 | Dm |
| N-glycan biosynthesis | 6937 | NP_014728 | OST3 | Sc |
| Proteasome | 2107 | NP_011748 | NAS6 | Sc |
| Neuroactive ligand-receptor interaction | 5254 | NP_499771 | 3O517 | Ce |
|  |  | NP_609037 | CG9543 | Dm |
| RNA polymerase | 14052 | NP_012950 | RPC37 | Sc |
|  |  | NP_524192 | Sin | Dm |

^1^While Nmd3p has previously been shown to mediate the translocation of the pre-60S ribosomal unit from the nucleus to the cytoplasm [60-62], its interactions with annotated mitochondrial proteins was unexpected. We speculate that this association could reflect a role for Nmd3p in accompanying ribosomal subunits to the outer membrane of the mitochondria, where the ribosomal complex could be translocated by the mitochondrial machinery (to eventually contribute to translation of the mitochondrial proteins).
