# Supplementary material for: Ulysses - an application for the projection of molecular interactions across species
Source: Genome Biol. 2005 Dec 2;6(12):R106. doi: 10.1186/gb-2005-6-12-r106 (PMC1414088; doi:10.1186/gb-2005-6-12-r106)
Supplement: Additional data file 2 — Double linkage criteria (see Table 5) revealed high confidence protein associations. Interacting partners 1 and 2 are listed with their human gene symbols and HomoloGene groups [file gb-2005-6-12-r106-S2.doc]

**Supplemental Table 2**. Previously known interactions. Human protein interaction predictions supported by redundant observations for homologous proteins in model organisms. Double linkage criteria (see Table 5) revealed high confidence protein associations. Interacting partners 1 and 2 are listed with their human gene symbols and HomoloGene groups.

| HomoloGene id 1 | Gene symbol 1 | HomoloGene 2 | Gene Symbol 2 |
| --- | --- | --- | --- |
| 5254  6462  3836  4028  20752  20752  20752  6230  5340  5837  5851  6781  6781  6781  9419  31505  5988  22239  728  730  6421  2080  2080  2080  2081  2084  2089  2090  2091  2097  4744  4744  2109  2110  4240  7157  2104  6885  2188  6288  6288  6730  2208  6517  4791  4856  6678  2512  6811  6120  21172  10129  3836  7140  6303  6476  2839  2901  20740  2155  5699 | COPE  CRNKL1  ERCC4  GTF2E1  H2AFE  H2AFJ  H2AFN  LSM1  LSM2  LSM4  LSM5  LSM7  LSM7  LSM7  LSM8  MAP2K2  NUP54  POLR1D  PPP3CA  PRIM1  PRPF19  PSMA1  PSMA1  PSMA1  PSMA2  PSMA5  PSMB3  PSMB4  PSMB5  PSMC3  PSMC4  PSMC4  PSMD12  PSMD13  PSMD14  PSMD6  PSMD7  RFC2  RFC3  RFC4  RFC4  RFC5  RPA1  RPLP0  RRAGB  RUVBL2  SF3B2  UBE2N  IMP4  IMP3  RAD50  FBXW7  ERCC4  TIPRL  VPS25  HKE2  NAPA  APPBP1  STAM  RAD51  SKP1A | 3218  4949  1501  1584  4355  4355  4355  5340  3381  6230  3381  5837  5851  5340  5340  21463  31711  3586  729  731  4949  2081  2082  2105  2086  2086  2093  2093  2081  2081  2097  2103  2110  2104  2110  2102  4240  6730  2187  2187  2188  6288  2209  777  6006  2749  22334  2513  4229  4229  4083  5699  325  3063  5239  1972  2791  2951  3457  5574  4877 | COPA  SF3A3  ERCC1  GTF2E2  NAP1L1  NAP1L1  NAP1L1  LSM2  SNRPD2  LSM1  SNRPD2  LSM4  LSM5  LSM2  LSM2  MAPK1  NUP62  POLR1C  PPP3R1  PRIM2A  SF3A3  PSMA2  PSMA3  PSMD8  PSMA7  PSMA7  PSMB7  PSMB7  PSMA2  PSMA2  PSMC3  PSMD4  PSMD13  PSMD7  PSMD13  PSMD3  PSMD14  RFC5  RFC1  RFC1  RFC3  RFC4  RPA2  RPLP1  RRAGC  RUVBL1  SF3B4  UBE2V2  MPHOSPH10  MPHOSPH10  MRE11A  SKP1A  XPA  PPP2CB  SNF8  PFDN5  STX16  UBE1C  HGS  UBE2I  SUGT1 |
